# Minimum-Integer Computation Finite Alphabet Message Passing Decoder: From Theory to Decoder Implementations towards 1 Tb/s

**DOI:** 10.3390/e24101452

**Published:** 2022-10-12

**Authors:** Tobias Monsees, Oliver Griebel, Matthias Herrmann, Dirk Wübben, Armin Dekorsy, Norbert Wehn

**Affiliations:** 1 Department of Communications Engineering, University of Bremen, 28359 Bremen, Germany; 2 Microelectronic Systems Design Research Group, TU Kaiserslautern, 67663 Kaiserslautern, Germany

**Keywords:** LDPC code, decoding, finite alphabet message passing, information bottleneck, implementation efficiency

## Abstract

In Message Passing (MP) decoding of Low-Density Parity Check (LDPC) codes, extrinsic information is exchanged between Check Nodes (CNs) and Variable Nodes (VNs). In a practical implementation, this information exchange is limited by quantization using only a small number of bits. In recent investigations, a novel class of Finite Alphabet Message Passing (FA-MP) decoders are designed to maximize the Mutual Information (MI) using only a small number of bits per message (e.g., 3 or 4 bits) with a communication performance close to high-precision Belief Propagation (BP) decoding. In contrast to the conventional BP decoder, operations are given as discrete-input discrete-output mappings which can be described by multidimensional LUTs (mLUTs). A common approach to avoid exponential increases in the size of mLUTs with the node degree is given by the sequential LUT (sLUT) design approach, i.e., by using a sequence of two-dimensional Lookup-Tables (LUTs) for the design, leading to a slight performance degradation. Recently, approaches such as Reconstruction-Computation-Quantization (RCQ) and Mutual Information-Maximizing Quantized Belief Propagation (MIM-QBP) have been proposed to avoid the complexity drawback of using mLUTs by using pre-designed functions that require calculations over a computational domain. It has been shown that these calculations are able to represent the mLUT mapping exactly by executing computations with infinite precision over real numbers. Based on the framework of MIM-QBP and RCQ, the Minimum-Integer Computation (MIC) decoder design generates low-bit integer computations that are derived from the Log-Likelihood Ratio (LLR) separation property of the information maximizing quantizer to replace the mLUT mappings either exactly or approximately. We derive a novel criterion for the bit resolution that is required to represent the mLUT mappings exactly. Furthermore, we show that our MIC decoder has exactly the communication performance of the corresponding mLUT decoder, but with much lower implementation complexity. We also perform an objective comparison between the state-of-the-art Min-Sum (MS) and the FA-MP decoder implementations for throughput towards 1 Tb/s in a state-of-the-art 28 nm Fully-Depleted Silicon-on-Insulator (FD-SOI) technology. Furthermore, we demonstrate that our new MIC decoder implementation outperforms previous FA-MP decoders and MS decoders in terms of reduced routing complexity, area efficiency and energy efficiency.

## 1. Introduction

Beyond 5G and 6G wireless communication systems, target peak data rates of 100 Gb/s to 1 Tb/s with processing latencies between 10–100 ns [1]. For such high data rate and low latency requirements, the implementation of a Forward Error Correction (FEC) decoder, which is one of the most complex and computationally intense components in the baseband processing chain, is a major challenge [2]. Low-Density Parity Check (LDPC) codes [3] are FEC codes with capacity approaching error correction performance [4] and are part of many communication standards, e.g., DVB-S2x, Wi-Fi, and 3GPP 5G-NR. In contrast to other competitive FEC codes, like Polar and Turbo codes, the decoding of LDPC codes is dominated by data transfers [2] making very high-throughput decoders in advanced silicon technologies challenging, especially from routing and energy efficiency perspectives. For example, in a state-of-the-art 14 nm silicon technology, the transfer of 8 bits on a 1 mm wire costs about 1 pJ, whereas the cost of an 8 bit integer addition is only 10 fJ, which is two orders of magnitude less than the wiring energy cost. During Message Passing (MP) decoding, two sets of nodes, the Check Node (CN) and Variable Node (VN), iteratively exchange messages over the edges of a bipartite graph (Tanner graph of the LDPC code). High-throughput decoding can be achieved by mapping the Tanner graph one-to-one onto hardware, i.e., dedicated processing units are instantiated for each node and the edges of the Tanner graph are hardwired. Unrolling and pipelining the decoding iterations can further boost the throughput towards 1 Tb/s [5], called unrolled full parallel (FP) decoders in the following. However, FP decoders imply large routing challenges, since every edge in the Tanner graph corresponds to $2\cdot I\cdot n_{E}$ wires, with *I* being the number of decoding iterations and $n_{E}$ being the quantization-width of the exchanged messages. Moreover, to enable good error correction performance, the Tanner graph exhibits limited locality and regularity, which makes efficient routing even more difficult. This problem is even exacerbated in advanced silicon technologies, as routing scales much worse than transistor density [6].

Finite Alphabet Message Passing (FA-MP) decoding has been investigated as a method to mitigate the routing challenges in FP LDPC decoders to reduce the bit-width, i.e., the quantization-width $n_{E}$, of the exchanged messages and, thus, the number of necessary wires [7,8,9]. In contrast to conventional MP decoding algorithms like the Belief Propagation (BP) and its approximations, i.e., Min-Sum (MS), Offset Min-Sum (OMS) and Normalized Min-Sum (NMS) [10], FA-MP use non-uniform quantizers and the node operations are derived by maximizing MI between exchanged messages. Nodes in state-of-the-art FA-MP decoders have to be implemented as Lookup-Tables (LUTs). Since the size of the LUT exponentially increases with the node degree and $n_{E}$, investigations were performed to decompose this multidimensional LUT (mLUT) into a chain or tree with only two-input LUTs (denoted as sequential LUT (sLUT) in this paper) yielding only a linear dependency of the node degree but at the cost of a decreased communications performance [11,12]. The Minimum-LUT (Min-LUT) decoder [13] approximates the CN update by a simple minimum search and can be implemented as Minimum-mLUT (Min-mLUT) or Minimum-sLUT (Min-sLUT), i.e., with mLUT or sLUT for VNs, respectively. Other approaches, e.g., Mutual Information-Maximizing Quantized Belief Propagation (MIM-QBP) [14,15,16] and Reconstruction-Computation-Quantization (RCQ) [17,18], are adding non-uniform quantizers and reconstruction mappings to the outputs and inputs of the nodes, respectively, and performing the standard functional operations inside the nodes, e.g., additions for VNs and minimum search for CNs. The reconstruction mappings generally increase the bit resolution required for node internal representation and processing. It can be shown that this approach is equivalent in terms of error correction performance compared to the mLUT, if the internal quantization after the reconstruction mapping is sufficiently large.

Based on the framework of MIM-QBP and RCQ, the proposed MIC decoder [19] realizes CN updates by a minimum search and VN updates by integer computations that are designed to realize the information maximizing mLUT mappings either exactly or approximately. In this paper, we provide more detailed explanations, extend the discussion to irregular LDPC codes and present a comprehensive implementation analysis. The new contributions of this paper (*Notation:* Random variables are denoted by sans-serif letters **x**, random vectors by bold sans-serif letters **x**, realizations by serif letters *x* and vector-valued realizations by bold serif letters ***x***. Sets are denoted by calligraphic letters 𝒳. The distribution $p_{x}\left(x\right)$ of a random variable x is abbreviated as p(x). x→y→z denotes a Markov chain, and R, Z, $F_{2}$ denotes the real numbers, integers and Galois field 2, respectively.) are summarized as follows:

- We provide a novel criterion for the resolution of internal node operations to ensure that the MIC decoder can always replace the information maximizing VN mLUT exactly;
- we show that this MIC decoder has the same communication performance compared to an MI maximizing Min-mLUT decoder;
- we make an objective comparison between different FA-MP decoder implementations (Min-mLUT, Min-sLUT, MIC) in an advanced silicon technology and compare them with a state-of-the-art MS decoder for throughput towards 1 Tb/s;
- we show that our MIC decoder implementation outperforms state-of-the-art FP decoders in terms of routing complexity, area efficiency and energy efficiency and enables the processing of larger block sizes in state-of-the-art FP decoders since the routing complexity is largely reduced.

The remainder of this paper is structured as follows: Section 2 reviews the system model, conventional decoding techniques for LDPC codes such as BP and NMS decoding, and Information Bottleneck (IB) based quantization. Section 3 describes the Min-mLUT and Min-sLUT decoder design for regular and irregular LDPC codes. In Section 4, we introduce the proposed MIC decoder and, in Section 5, we discuss the MIC decoder implementation along with a detailed comparison with state-of-the-art FP MP decoders. Finally, Section 6 concludes the paper.

## 2. Preliminaries

This section briefly reviews the transmission model, conventional decoding techniques for LDPC codes, and the quantizer design based on IB.

### 2.1. Transmission Model

**Figure 1:**
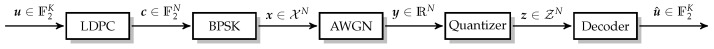
Transmission model for transmission of LDPC encoded messages over an AWGN channel with quantization prior to FEC decoding.

The transmission model is shown in Figure 1. An information word $u\in F_{2}^{K}$ is encoded into the codeword $c\in F_{2}^{N}$ via a binary LDPC code [3] of rate $R=\frac{K}{N}$. The Binary Phase Shift Keying (BPSK) modulated vector x=1−2c is transmitted over an Additive White Gaussian Noise (AWGN) channel leading to the received vector $y\in R^{N}$ given by y=x+n with AWGN ***n*** of variance $\sigma_{n}^{2}$. A particular LDPC code is defined via a sparse parity check matrix $H\in F_{2}^{M\times N}$. The Tanner graph [20] of an LDPC code is a visual representation of its parity check matrix ***H*** and consists of a CN for each parity check equation $\chi_{m}$ with m=1,...,M and a VN for each codebit $c_{n}$ with n=1,...,N. An edge connects VN *n* and CN *m* if and only if $H_{m,n}=1$. The degree of a node is determined by the number of connected edges. Furthermore, the fraction of edges that is connected to a node of a specific degree is characterized by the edge-degree distributions

(1) $$\lambda \left(\xi \right)=\sum_{d_{V}\in D_{V}}\lambda_{d_{V}}\xi^{d_{V}-1}\;\mathrm{and}\;\rho \left(\xi \right)=\sum_{d_{C}\in D_{C}}\rho_{d_{C}}\xi^{d_{C}-1}\;$$

where $\lambda_{d_{V}}$ is the fraction of edges that are connected to VNs of degree $d_{V}\in D_{V}$, and $\rho_{d_{C}}$ denotes the fraction of edges that is connected to CNs of degree $d_{C}\in D_{C}$.

### 2.2. Iterative Decoding via Belief-Propagation (BP)

**Figure 2:**
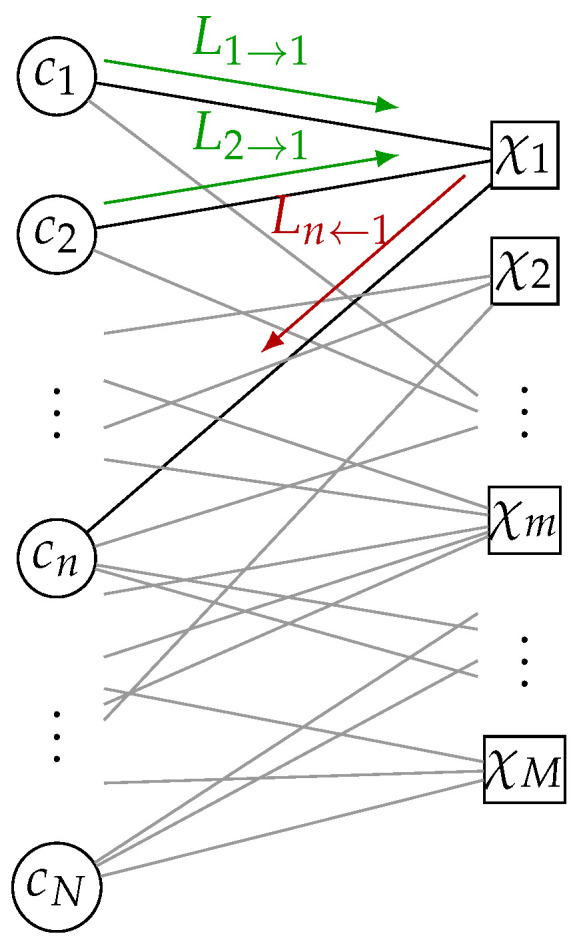
Illustrative example of a CN update on a Tanner graph. The CN $\chi_{1}$ generates the CN-to-VN message $L_{n\leftarrow 1}$ for the VN $c_{n}$ based on the VN-to-CN messages $L_{1\to 1}$ and $L_{2\to 1}$ from VN $c_{1}$ and $c_{2}$, respectively.

LDPC codes are usually decoded by iterative BP, where *extrinsic information* for each codebit $c_{n}$ is propagated along the edges of the resulting Tanner graph. Figure 2 shows the CN $\chi_{1}$ that generates *extrinsic information* for the VN $c_{n}$ by processing the incoming Variable Node to Check Node (VN-to-CN) messages from the other VNs connected to CN $\chi_{1}$, i.e., $c_{1}$ and $c_{2}$. For BP decoding, we define the Variable Node to Check Node (VN-to-CN) messages as $L_{n\to m}\in R$ and the Check Node to Variable Node (CN-to-VN) messages as $L_{n\leftarrow m}\in R$. In the first iteration, all messages are initialized by the channel LLRs

(2) $$L_{n\to m}^{\left(0\right)}=L\left(y_{n}\right)=\frac{2}{\sigma_{n}^{2}}y_{n}\;.$$

In iteration *i*, a CN *m* generates *extrinsic information* for the connected VNs $n\in M_{m}$ via the box-plus operation

(3) $$L_{n\leftarrow m}^{\left(i\right)}=2\left(\prod_{j\in M_{m}\backslash n}\tanh\left(\frac{1}{2}L_{j\to m}^{\left(i-1\right)}\right)\right)\;,\;\forall n\in M_{m}\;.$$

In case of Normalized Min-Sum (NMS) decoding, the CN update (3) is approximated by

(4) $$L_{n\leftarrow m}^{\left(i\right)}\approx \gamma \left(\prod_{j\in M_{m}\backslash n}\mathrm{sign}\left(L_{j\to m}^{\left(i-1\right)}\right)\right)\min_{j\in M_{m}\backslash n}\left|L_{j\to m}^{\left(i-1\right)}\right|\;,\;\forall n\in M_{m}\;.$$

where γ is the normalization factor. In the case of γ=1, (4) is the CN update of the MS decoder.

In similar fashion, a VN *n* generates *extrinsic information* for the connected CNs $m\in N_{n}$ by adding the corresponding LLRs

(5) $$L_{n\to m}^{\left(i\right)}=L\left(y_{n}\right)+\sum_{v\in N_{n}\backslash m}L_{n\leftarrow v}^{\left(i\right)}\;,\;\forall m\in N_{n}\;.$$

The final bit decision ${\hat{c}}_{n,\mathrm{BP}}^{\left(i\right)}$ at iteration *i* is determined by

(6) $${\hat{c}}_{n,\mathrm{BP}}^{\left(i\right)}=\frac{1}{2}\left(1-\mathrm{sign}\left(L\left(y_{n}\right)+\sum_{v\in N_{n}}L_{n\leftarrow v}^{\left(i\right)}\right)\right)\;.$$

### 2.3. Information Bottleneck Based Quantizer Design

**Figure 3:**
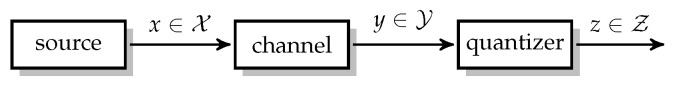
Considered system model for quantizer design.

For the design of our proposed MIC decoder, we utilize MI maximizing quantization to design an information optimized processing chain that uses only quantizer labels instead of real valued representations [12]. To that end, we first review the principle idea of the MI based quantizer design approach. The considered system model is visualized in Figure 3. The observed signal y∈Y is mapped to a compressed representation z∈Z via the scalar quantization function Q:Y→Z. The objective is to find a quantizer function $Q^{★}$ that maximizes MI I(x;z) between the relevant source x∈X and the quantizer output Q(y)=z∈Z under the condition that the three random variables form a Markov chain x→y→z. Given the joint distribution p(x,y)=p(y|x)p(x), the mapping of the information maximizing quantizer $Q^{★}$ is determined by solving the optimization problem

(7) $$Q^{★}=\underset{Q}{\mathrm{argmax}}\;I\left(x;z\right)\;s.t.\;|Z|=2^{n_{Q}}<\left|Y\right|\;$$

where the number of possible quantizer outputs is set to $2^{n_{Q}}$. The optimization problem in (7) is a special case of the Information Bottleneck Method (IBM) [12,21,22,23]. The optimal solution is a deterministic quantization function where the conditional probability of the quantizer output *z* given the relevant source *x* is

(8) $$p\left(z|x\right)=\sum_{y\in Y_{z}}p\left(y|x\right)\;$$

with $Y_{z}=\left\{y\in Y\;|\;Q^{★}\left(y\right)=z\right\}$ as the set of observed signals *y* that are mapped to one specific quantizer output *z*. Since the maximum of (7) depends only on the cardinality of Z, we utilize a convenient signed integer based representation $Z=\{-\frac{2^{n_{Q}}}{2},...,-1,1,...,\frac{2^{n_{Q}}}{2}\}$ that simplifies the MIC decoder processing. For the special case where the relevant source x is a binary random variable (i.e., |X|=2), the algorithm that finds the optimal quantizer via dynamic programming has been derived in [24]. We denote the LLRs of the quantizer output z∈Z by

(9) $$L\left(z\right)=\log\left(\frac{p(z|x=+1)}{p(z|x=-1)}\right)\;.$$

An important property of the MI maximizing quantizer for binary input is that any two different sets of LLRs $L_{z^{\prime }}=\left\{L\left(y\right)\;|\;y\in Y_{z^{\prime }}\right\}$ and $L_{z^{\prime \prime }}=\left\{L\left(y\right)\;|\;y\in Y_{z^{\prime \prime }}\right\}$ for $z^{\prime },z^{\prime \prime }\in Z$ and $z^{\prime }\neq z^{\prime \prime }$ are separated by a single threshold [19,24,25]. This property will be exploited in the design of the MIC decoder in Section 4.

## 3. LUT Decoder Design

This section describes the design of the LUT decoder that is optimized via Discrete Density Evolution (DDE) [11] to maximize *extrinsic information* between the codebits and its messages, under the assumption that the Tanner graph is cycle free. In contrast to the BP algorithm, the LUT is optimized to process the quantizer labels *z* in (7) directly and the bit resolution of the message exchange on the Tanner graph is limited to $n_{E}$ bits, e.g., 3 or 4 bits. Furthermore, we exploit the signed integer-based representation to simplify the CN update by using the label-based minimum search [13]. In the Min-mLUT decoder design, the VN update functions are optimized to maximize MI. For the Min-sLUT decoder design, the VN update is decomposed into a sequence of two-dimensional updates that generally results in a MI loss compared to the Min-mLUT decoder design.

**Figure 4:**
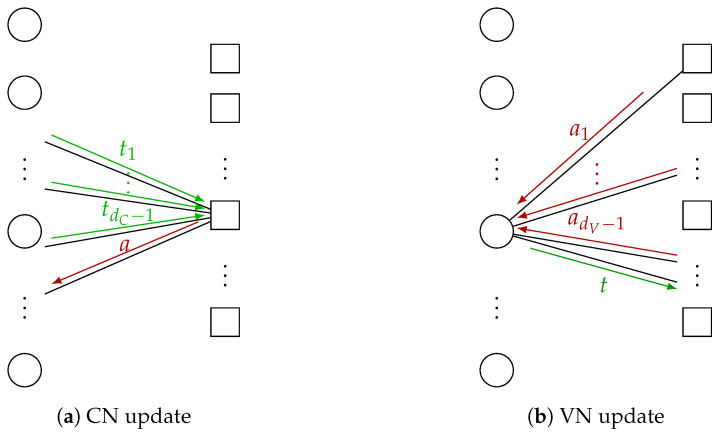
Illustrative example for generation of extrinsic information in case of LUT decoding using discrete messages. (**a**) visualizes a CN that generates the CN-to-VN message *a* based on incoming VN-to-CN messages $t_{1},...,t_{d_{C}-1}$. In (**b**), a VN generates the VN-to-CN message *t* based on incoming CN-to-VN messages $a_{1},...,a_{d_{V}-1}$.

In the following, we review the calculation of the CN and VN distributions for each iteration that are required for the design of the MI maximizing VN update. As illustrated in Figure 4, we omit the iteration index *i* and consider messages of an arbitrary CN and VN for CN degrees $d_{C}\in D_{C}$ and VN degrees $d_{V}\in D_{V}$ to calculate the distributions that are required for the Min-mLUT design.

### 3.1. Check Node LUT Design

The LUT decoder design is based on discrete alphabets Z, T and A for the channel information, the VN-to-CN and the CN-to-VN messages, respectively. For the first iteration, the VN-to-CN messages $t_{j}$ for $j=1,...,d_{V}-1$ are initialized by the signed integer valued channel information, i.e., $t_{j}=z_{j}\in Z$. The distribution of the $d_{C}-1$ VN-to-CN messages $t=\left[t_{1},...,t_{d_{C}-1}\right]\in T^{\left(d_{C}-1\right)}$ and an arbitrary codebit *c* of a check equation χ is [11]

(10) $$p_{d_{C}}\left(t|c\right)={\left(\frac{1}{2}\right)}^{d_{C}-2}\sum_{b:⨁b=c}\prod_{j=1}^{d_{C}-1}p\left(t_{j}|b_{j}\right)\;$$

with $⨁b=b_{1}⊕...⊕b_{d_{C}-1}$ as the modulo 2 sum of connected codebits. The VN-to-CN messages $t_{j}$ are processed by a CN update function that generates quantized output messages a∈A that are represented only by $n_{E}$ bits.

Given the distribution in (10), the CN update (We keep the node degrees $d_{C}$ or $d_{V}$ as index of random variables to indicate that the distribution changes with the corresponding degrees.) $f_{d_{C}}\left(t_{d_{C}}\right)=a_{d_{C}}$ that maximizes MI is determined by the solution of the quantization problem for binary input ($c\to t_{d_{C}}\to f_{d_{C}}\left(t_{d_{C}}\right)=a_{d_{C}}$)

(11) $$f_{d_{C}}^{\mathrm{MI}}=\underset{f_{d_{C}}}{\mathrm{argmax}}\;\;I\left(c;t_{d_{C}}\right)\;\;s.t.\;\;\left|A\right|=2^{n_{E}}\;\mathrm{for}\;\;d_{C}\in D_{C}\;.$$

As discussed in Section 2.3, the optimal solution of (11) is found via dynamic programming.

**Figure 5:**
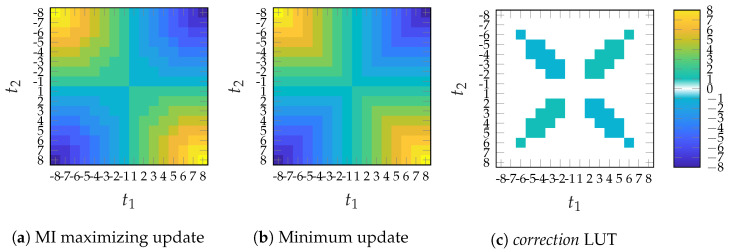
Graphical representation of a discrete CN update using $n_{E}=4$ bit input messages $t_{1}$ and $t_{2}$ and a color-coded output message a∈A={−4,...,−1,1,...,4}. Subfigure (**a**) shows the MI maximizing update $f_{3}^{\mathrm{MI}}\left(t_{1},t_{2}\right)$ and subfigure (**b**) the minimum update $f_{3}^{\min}\left(t_{1},t_{2}\right)$. The difference $f_{3}^{\mathrm{MI}}\left(t_{1},t_{2}\right)-f_{3}^{\min}\left(t_{1},t_{2}\right)$ in subfigure (**c**) contains only a few non-zero elemets and can be interpreted as a *correction* LUT.

However, we utilize the minimum update [13] as a CN update for all iterations as an approximation of the MI maximizing CN update in (11). We observed that the output of the minimum update is quite close to the optimal IB update. As visualized for a degree 3 CN in Figure 5, the difference between the optimal IB CN and the minimum update can be interpreted as an additive *correction* LUT where only a small fraction of entries are nonzero. For the label-based minimum search, the CN update rule reads

(12) $$a=\;f_{d_{C}}^{\min}\left(t\right)\;=\;\left(\prod_{j=1}^{d_{C}-1}\mathrm{sign}\left(t_{j}\right)\right)\min\{|t_{1}\left|,...,\right|t_{d_{C}-1}\left|\right\}\;.$$

If the CN update function is given, the conditional distribution of the CN-to-VN messages a∈A=T is

(13) $$p_{d_{C}}\left(a|c\right)=\sum_{t\in T^{\left(d_{C}-1\right)}\;:\;f_{d_{C}}^{\min}\left(t\right)=a}p_{d_{C}}\left(t|c\right)\;\mathrm{for}\;\;d_{C}\in D_{C}\;.$$

In the design via DDE, the connections between VNs and CNs are considered on average by the degree distribution [26]. Hence, the design considers only the marginal CN-to-VN message distribution p(a|c) that includes averaging over all possible CN degrees by

(14) $$p\left(a|c\right)=\sum_{d_{C}\in D_{C}}p_{d_{C}}\left(a|c\right)\rho_{d_{C}}\;.$$

### 3.2. Variable Node LUT Design

For designing the VN update, we require the joint distribution of the discrete channel information z∈Z together with the CN-to-VN messages $a_{m}\in A$ combined in $a=\left[z,a_{1},...,a_{d_{V}-1}\right]\in Z\times A^{d_{V}-1}=V$ and a codebit *c* [11]

(15) $$p_{d_{V}}\left(a|c\right)=p\left(z|c\right)\prod_{m=1}^{d_{V}-1}p\left(a_{m}|c\right)\;$$

where $p\left(a_{m}|c\right)=p\left(a|c\right)$ for $m=1,...,d_{V,\max}-1$ and V is the set of all possible states of the vector *a*, i.e., $\left|V\right|=2^{n_{Q}+\left(d_{V}-1\right)n_{E}}$. Given the distribution (15), the individual degree-dependent VN update $g_{d_{V}}\left(a_{d_{V}}\right)=t_{d_{V}}$ that maximizes MI $I(c;t_{d_{V}})$ is determined as the solution of the optimization problem ($c\to a_{d_{V}}\to g_{d_{V}}\left(a_{d_{V}}\right)=t_{d_{V}}$)

(16) $$g_{d_{V}}^{\mathrm{MI}}=\underset{g_{d_{V}}}{\mathrm{argmax}}\;\;I\left(c;t_{d_{V}}\right)\;\;s.t.\;\;\left|T\right|=2^{n_{E}}\;\mathrm{for}\;\;d_{V}\in D_{V}\;.$$

The parameter $n_{E}$ defines the bit-width of the messages exchanged between VN and CN and controls the complexity of the message exchange. The optimization problem in (16) is the channel quantization problem for binary input (Section 2.3). The optimal solution is a deterministic input–output relation that can be stored as a $d_{V}$ dimensional LUT with $2^{n_{Q}+\left(d_{V}-1\right)n_{E}}$ entries, e.g., for $d_{V}=6$ and $n_{E}=n_{Q}=4$, we have approximately 16.8 million entries. Furthermore, the communication performance can be increased by considering the degree distribution in the design of the node updates [13,26]. The gain in communication performance generally depends on the degree distribution and the message resolution $n_{E}$ [13]. However, a comparison of the different design approaches in [13,26] is beyond the scope of this paper. The distribution of the VN-to-CN messages for the next iteration in (10) is

(17) $$p_{d_{V}}\left(t|c\right)=\sum_{a\in V\;:\;g_{d_{V}}^{\mathrm{MI}}\left(a\right)=t}\;p\left(a|c\right)\;\;\mathrm{for}\;\;d_{V}\in D_{V}\;.$$

Again, the marginal distribution is determined by averaging over all possible VN degrees, i.e.,

(18) $$p\left(t_{j}|c_{j}\right)=p\left(t|c\right)=\sum_{d_{V}\in D_{V}}p_{d_{V}}\left(t|c\right)\lambda_{d_{V}}\;,\;\mathrm{with}\;j=1,...,d_{C,\max}\;.$$

In case of a regular LDPC code, there is only one possible degree for all VNs and CNs, i.e., the summation term in (14) and (18) vanishes but all other steps remain the same.

For the design of the MI maximizing Min-mLUT decoder, we start with an initial VN-to-CN distribution $p(t_{j}|c_{j})$ and iterate over (10), (13)–(18) and declare convergence if I(c;t) approaches the maximum value of one bit for binary input after *I* number of iterations.

### 3.3. Sequential LUT Design

For the sequential design approach sLUT, the node update is split into a sequence of degree two updates that are optimized independently to maximize MI. This approach serves as an approximation of the mLUT design described in Section 3.2 and reduces the number of possible memory locations within each update. In general, multidimensional optimization without decomposition conserves more MI compared to a design that decomposes the optimization problem into a sequence of two-dimensional updates [11,12] or more general nested tree decompositions [13].

## 4. Minimum-Integer Computation Decoder Design

**Figure 6:**
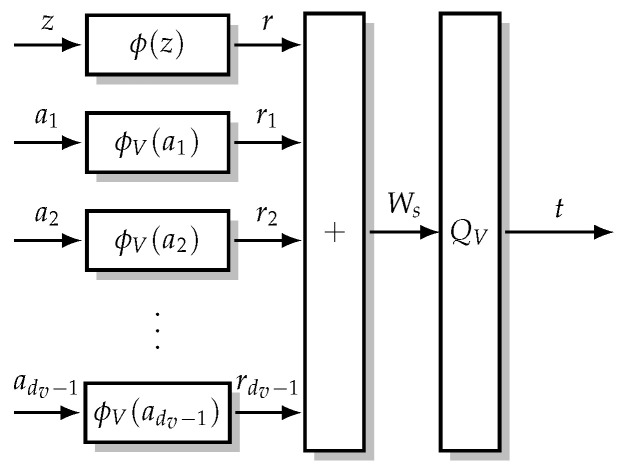
VN update for computational domain framework [14,16]. The $n_{Q}$-bit channel information z∈Z and the $n_{E}$-bit CN-to-VN messages $a_{1},...,a_{d_{V}-1}\in A$ are transformed to $n_{R}$-bit signed integers. This transformation generally increases the required bit resolution for the representation, i.e., $n_{R}\geq n_{Q}$ and $n_{R}\geq n_{E}$. The internal signed integers are summed and quantized back into a $n_{E}$-bit VN-to-CN message t∈T.

The MI maximizing Min-mLUT decoder realizes the discrete VN updates by LUTs with $2^{n_{Q}+\left(d_{V}-1\right)n_{E}}$ entries leading to prohibitively large implementation complexity. Nevertheless, determining these multidimensional LUTs in the laboratory is feasible with sufficient computing resources. Thus, the idea is to search for the MI maximizing mLUTs but implement the corresponding discrete functions by relatively simple operations in order to avoid performance degradations. As visualized in Figure 6, the computational domain framework [14,16] replaces the VN update by an operation that is decomposed into

(i) mappings $\phi_{V}$ and ϕ of the $n_{E}$-bit CN-to-VN messages $a_{m}$ and $n_{Q}$-bit channel information *z* into *node internal* $n_{R}$-bit signed integers with $n_{R}\geq n_{E}$ and $n_{R}\geq n_{Q}$, respectively;
(ii) execution of integer additions for $n_{R}$-bit signed integers;
(iii) threshold quantization $Q_{V}$ to $n_{E}$ bits determining the VN-to-CN message *t*.

For the MIC decoder design, we derive a criterion for sufficient internal node resolution $n_{R}$ such that the mLUT mapping is replaced exactly. Note that the information maximizing mLUT is generated offline and is replaced by an integer function that replaces its functionality exactly or approximately during execution.

To keep the notation simple, we omit the dependency on the iteration index *i* and node degree in this section.

### 4.1. Equivalent LLR Quantizer

To motivate the integer calculation of the MIC approach, we review the connection between the equivalent LLR quantizer and the VN update of the BP algorithm. Analogous to the VN update of the BP algorithm in (5), the LLR of the combined message vector a∈V equals the addition of the LLRs of the channel output *z* and of the individual messages $a_{m}$, i.e., for every possible combination a∈V, the LLR of the combined message is

(19) $$L\left(a\right)\;=\;\log\;\left(\frac{p(a|c=0)}{p(a|c=1)}\right)\;=\;L\left(z\right)\;+\;\sum_{m=1}^{d_{V}-1}\;L\left(a_{m}\right)\;.$$

The LLRs $L(a_{m})$ of the individual messages are determined by (14) during DDE. As described in Section 2.3, the information maximizing quantizer for binary input separates the LLR L(a) by using a |T|−1 threshold quantizer $Q_{L}:R\to T$, i.e., the relation

(20) $$t=g^{\mathrm{MI}}\left(a\right)=Q_{L}\left(L(a)\right)=Q_{L}\left(\;L\left(z\right)\;+\;\sum_{m=1}^{d_{V}-1}\;L\left(a_{m}\right)\right)$$

can be determined that achieves the same output as the information optimal mLUT in (16). However, to ensure that (20) produces the same output as the information optimal mLUT, calculations over real numbers are required. In the next subsection, we show that we can exploit (20) to find a calculation that requires only a finite resolution. We also provide a condition to limit the resolution that is required for exact calculation of the information optimal mLUT.

### 4.2. Computations over Integers

The VN update structure using the computational domain framework is visualized in Figure 6. As suggested by [14,16], a possible choice for the integer mappings $\phi_{v}\left(m\right)$ and $\phi_{ch}\left(z\right)$ is given by scaling and rounding the corresponding LLRs L(m) and L(z), respectively. In addition to [14,16], we provide further insights on the optimal choice of the scaling factor based on the relation between the VN update of the BP algorithm and the MI maximizing quantizer design. More precisely, based on the established relation in (20), we define an integer mapping for the channel information *z* and the CN-to-VN messages $a_{m}$ in order to replace the computations over real numbers by computations over signed integers (With ⌊·⌉ as round to nearest integer (away from 0 if fraction part is .5))

(21) $$g^{\mathrm{MIC}}\left(a\right)=Q_{V}\left(W_{s}\left(a\right)\right)=Q_{V}\left(\underset{r=\phi (z)\in R}{\underset{︸}{sL(z)}}+\sum_{m=1}^{d_{V}-1}\underset{r_{m}=\phi_{V}\left(a_{m}\right)\in R_{V}}{\underset{︸}{sL(a_{m})}}\right)\;=\;Q_{V}\left(r+\sum_{m=1}^{d_{V}-1}r_{m}\right)\;.$$

Compared to (20), the LLRs L(z) and $L(a_{m})$ have been multiplied by a non-negative scaling factor *s* and quantized to the next $n_{R}$-bit signed integer *r* and $r_{m}$, respectively. Subsequently, the sum of integers is limited again to $n_{E}$ bits by threshold quantizer $Q_{V}$. We can interpret the scaling and rounding operation also directly as a mapping of signed integer messages *z* and $a_{m}$ to $n_{R}$-bit signed integer messages

(22) $$r=\phi \left(z\right)\in R\;\mathrm{and}\;r_{m}=\phi_{V}\left(a_{m}\right)\in R_{V}$$

that requires $n_{R}$ bits for the representation, depending on the scaling factor *s*.

In the following, we show that we can always find a threshold quantizer $Q_{V}:W\to T$ that maps the summation $W_{s}\left(a\right)$ into a VN-to-CN message t∈T that is identical to the VN-to-CN message of the information optimal VN update in (20), i.e., $t=g^{\mathrm{MIC}}\left(a\right)=g^{\mathrm{MI}}\left(a\right)$. First, we consider the set of messages $A_{t}=\left\{a\in V:g^{\mathrm{MI}}\left(a\right)=t\in T\right\}$ that are mapped into a specific output *t* via the information maximizing VN update $g^{\mathrm{MI}}\left(a\right)$ in (16). Thus, we can identify a corresponding set of integers $W_{t}\;=\;\left\{W_{s}\left(a\right)\;\in \;W:a\;\in \;A_{t}\right\}$. By varying the scaling factor *s*, we can always find a scaling value $s^{★}\leq \frac{d_{V}}{\Delta_{\min}}$ such that the sets of integer values $W_{t}$ for all t∈T are *non-overlapping intervals*, i.e.,

(23) $$\left[D_{t^{\prime }},E_{t^{\prime }}\right]\cap \left[D_{t^{\prime \prime }},E_{t^{\prime \prime }}\right]=\emptyset \;\forall t^{\prime \prime },t^{\prime }\in T\;,$$

with $D_{t}=\min W_{t}$ and $E_{t}=\max W_{t}$. Condition (23) ensures that any two different clusters $t^{\prime }$ and $t^{\prime \prime }$ can be separated by a simple threshold operation. The value $\Delta_{\min}$ is the minimum separation between the LLRs L(a) of the elements of any two neighbouring clusters in (20) and is always larger than zero since $Q_{L}$ is a threshold quantizer. If we consider a scaled version of the LLRs sL(a) with any real valued scaling factor s>1, we can always find a threshold quantizers $Q_{L,s}$ that achieves the same output as the information optimal mLUT. Scaling the LLRs L(a) by a factor of $\frac{d_{V}}{\Delta_{\min}}$ ensures that the minimum separation between any two neighbouring clusters is $d_{V}$. Since the influence of the rounding operation can be bounded by $-\frac{d_{V}}{2}\leq W_{s}\left(a\right)-sL\left(a\right)<\frac{d_{V}}{2}$, scaling with a factor of at least $\frac{d_{V}}{\Delta_{\min}}$ ensures that any two neighbouring clusters $W_{t}$ and $W_{t+1}$ are separated by at least one integer and, thus, condition (23) is satisfied. Hence, we can always find a corresponding integer function $g^{\mathrm{MIC}}\left(a\right)$ in (21) that generates exactly the same output as $g^{\mathrm{MI}}\left(a\right)$ in (20).

Furthermore, an approximate integer calculation is found if the integer valued range of ϕ and $\phi_{V}$ are limited to $n_{R}$-bits

(24) $$\max_{z}\;|\phi \left(z\right)|<2^{n_{R}}<2^{n_{R}^{★}}\;\;,\;\max_{a}\;\left|\phi_{V}\left(a\right)\right|<2^{n_{R}}<2^{n_{R}^{★}}\;$$

where $n_{R}^{★}=\left\lceil \log_{2}\left(s^{★}L_{\max}\right)\right\rceil +1$ is the bit resolution that is required for exact representation if the largest magnitude of the individual LLRs in (20) is $L_{\max}$. If condition (23) is not fulfilled, we select the output cluster that maximizes MI. If (24) is satisfied, the required bit resolution of the summation $W_{s}\left(a\right)$ in (21) is limited by

(25) $$n_{W}=\left\lceil \log_{2}\left(d_{V}\left(2^{\left(n_{R}-1\right)}-1\right)\right)\right\rceil +1\;.$$

To consider the influence of this new mapping in the design of subsequent iterations, we also update the VN-to-CN distribution in (17).

We note that the MIC design approach can also be applied for the design of CN operations and can also be used to generate exact or approximate representations of nested tree decompositions similar to the sLUT method. However, the corresponding investigations are beyond the scope of this paper.

#### Illustrative Example for MIC Calculations

**Figure 7:**
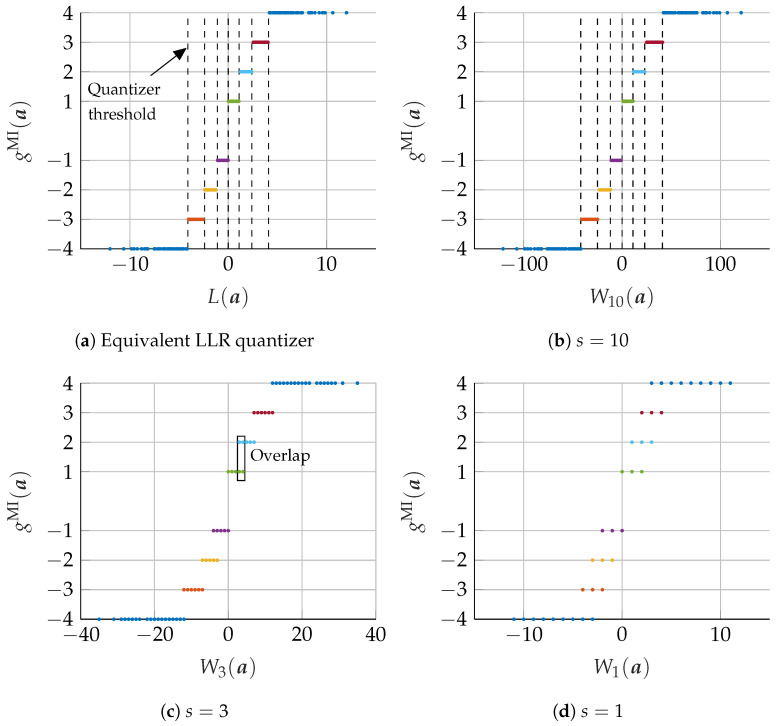
Visualization of the relationship between the result of the calculation in the computational domain and the assignment to mutual information maximizing mLUT mapping. Subfigure (**a**) shows the addition the real valued LLRs of (20) on the *x*-axis and the mutual information maximizing mLUT assignment of (16) on the *y*-axis. In Subfigure (**b**–**d**), the values on the *x*-axis are replaced by the corresponding integer additions of (21) for different scaling factors s∈{1,3,10}.

To illustrate the proposed MIC approach, we consider the design of a VN node update for a ($d_{V}$ = 3, $d_{C}$ = 6) regular LDPC codes at iteration i=1 with design $E_{b}/N_{0}=2.5$ dB. Figure 7a shows the equivalent LLR quantizer (20) with $2^{n_{E}}$*non-overlapping* clusters on the real number line, i.e., T={−4,...,−1,1,...,4}. e.g., all LLRs L(a) between 0 and 1.1 are mapped into cluster t=1. The threshold values are shown by dashed lines in Figure 7a. Additionally, Figure 7b–d show the output of the integer addition in (21) on the *x*-axis and the output clusters of the optimal mLUT on the *y*-axis if the scaling factor is set to s=10, s=3, and s=1, respectively.

In the case of s=10, all output clusters are separated by using seven integer thresholds, which are indicated by dashed lines in Figure 7b. In this case, the integer computation fully replaces the original mLUT functionality by using only signed integers of low-range. To clarify the example, the numeric values of the corresponding LLRs and integer mappings of (19) and (21) for s=10 are shown in Table 1. For example, the quantized receive message z=2 corresponds to an LLR of L(z)=1.56 leading to the $n_{R}$-bit signed integer message r=ϕ(z)=15.6=16. After summation of *r* and $r_{m}$, all results $12\leq W_{10}\left(a\right)\leq 23$ are again mapped back to the $n_{E}$ message t=2.

For s=3 and s=1, the integer range is further reduced, but the original mLUT functionality cannot be represented exactly since some integer additions are mapped to more than one output cluster of the original mLUT (e.g., some values of $W_{s}\left(a\right)$ are mapped into cluster t=1 and t=2 as highlighted in Figure 7c). If some values of $W_{s}\left(a\right)$ are assigned to more than one cluster of the information maximizing mLUT mapping, a merging of these values into a single cluster is required. This merging generally leads to an inevitable loss of information. In order to find a corresponding threshold quantizer for this case, we select the output cluster that minimizes the information loss under the condition that (23) is fulfilled.

### 4.3. FER Results

**Figure 8:**
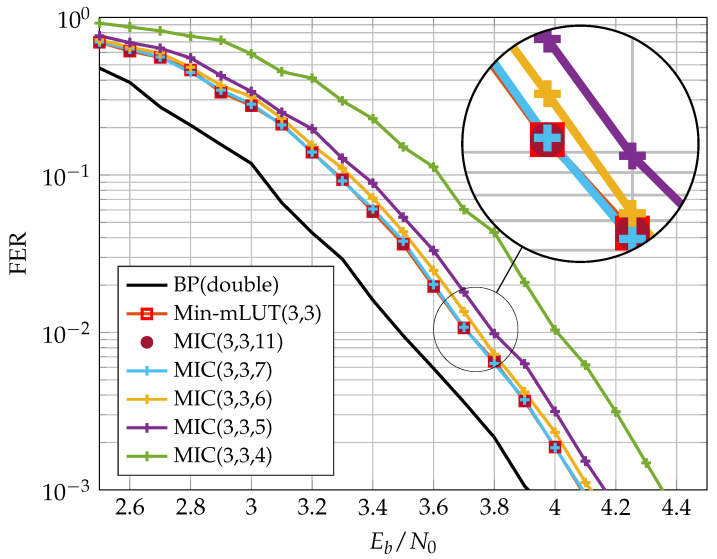
FER performance of $n_{E}=n_{Q}=3$ bit Min-mLUT and MIC decoders using different internal message resolutions $n_{R}$ for VN update.

In this section, we discuss the communication performance of the proposed MIC decoder for an irregular LDPC code from the 802.11n standard [27] of length N=648 with rate R=0.75 and edge degree distributions $\lambda \left(\xi \right)=0.2083\xi^{1}+0.3333\xi^{2}+0.25\xi^{3}+0.2083\xi^{5}$ and $\rho \left(\xi \right)=\frac{1}{3}\xi^{13}+\frac{2}{3}\xi^{15}$. The realization of the MIC decoder is characterized by three quantization parameters and specified by MIC($n_{E},n_{Q},n_{R}$). In contrast, the Min-mLUT decoder with label based minimum operation as CN update has only two parameters and is denominated by Min-mLUT($n_{E},n_{Q}$). Figure 8 shows the Frame Error Rate (FER) performance of Min-mLUT and MIC for $n_{E}=n_{Q}=3$ and I=10 iterations, but varying resolution of internal messages $n_{R}\in \left\{4,5,6,7,11\right\}$ for MIC.

The BP decoder with double precision serves as our benchmark simulation. The Min-mLUT decoder with $n_{E}=n_{Q}=3$ bit quantization for the message exchange and channel information results in a minor performance degeneration of only 0.2 dB at a FER of $10^{-3}$ w.r.t. the benchmark simulation. In comparison, the proposed MIC decoder that replaces the VN update of the Min-mLUT decoder by using the computational domain framework with internal messages of size $n_{R}=4$ results in a loss of 0.25 dB compared to the Min-mLUT decoder. The performance gain of the MIC decoder by using $n_{R}=5$ compared to $n_{R}=4$ is around 0.1 dB. The MIC decoder with $n_{R}=7$ has basically identical FER performance compared to the Min-mLUT decoder. If $n_{R}=11$, the MIC decoder represents the mLUT functionality exactly by meeting the criterion (23), but the gain in communication performance compared to the MIC decoder with $n_{R}=7$ is negligible. Additionally, MIC decoding does not require LUTs with up to 262k entries for each iteration.

## 5. Finite Alphabet Message Passing (FA-MP) Decoder Implementation

**Figure 9:**
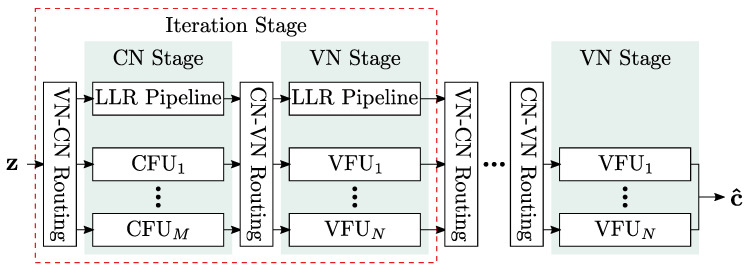
Unrolled full-parallel decoding architecture.

In this section, we investigate the implementation complexity of different LUT-based FA-MP decoders in terms of area, throughput, latency, power, area efficiency, and energy efficiency and compare them with a state-of-the-art Normalized Min-Sum (NMS) decoder. As already stated, we focus on unrolled full parallel (FP) decoder architectures that enable throughput towards 1 Tb/s. The architecture template is shown in Figure 9. Input to the decoder are compressed messages *z* from the channel quantizer. The decoder uses two-phase decoding. Hence, each iteration consists of two stages: one stage comprises *M* Check Node Functional Units (CFUs) and the second stage *N* Variable Node Functional Units (VFUs). The stages are connected by hardwired routing networks, which implement the edges of the Tanner graph. Since the decoding iterations are unrolled, the decoder consists of 2·I stages. Deep pipelining is applied to increase the throughput. For more details on this architecture, the reader is referred to [5].

In FP decoders that use the NMS algorithm, node operations are implemented as additions and minimum searches on uniformly quantized messages [5]. In contrast, node functionality in Finite Alphabet (FA) decoders is implemented as LUTs. Implementing a single LUT as memory is impractical in Application-Specific Integrated Circuit (ASIC) technologies since the area and power overhead would be too large. Hence, a single LUT is transformed into $n_{E}$ Boolean functions $b:B^{inp}\to B$ with inp being the number of inputs of the LUT, which is the node degree multiplied by $n_{E}$. *b* can consist of up to $2^{inp}$ product terms if *b* is represented in sum-of-product form. State-of-the-art logic synthesis tools try to minimize *b* such that it can be mapped onto a minimum number of gates. Despite this optimization, the resulting logic can be very large for higher node degrees and/or $n_{E}$, making this approach unsuitable for efficient FP decoder implementation. It was shown in [7] that the mLUT can be decomposed into a set of two-input sLUTs arranged in a tree structure, which largely reduces the resulting logic at the cost of a small degradation in error correction performance. To compare these approaches with our new decoder, we implemented four different types of FP decoders:

- NMS decoder with extrinsic message scaling factor of 0.75;
- Two LUT-based decoders: in these decoders, we implemented the VN operation by LUTs and the CN operations by a minimum search on the quantized messages. The latter corresponds to the CN Processor implementation of [7]. The LUTs are implemented either as a single LUT (mLUT), or as a tree of two-input LUTs (sLUT);
- Our new MIC decoder in which the VN is replaced by the new update algorithms, presented in the previous section.

For MIC and LUT based decoders, we investigated message quantization $n_{E}=3$ and $n_{E}=4$. The reference is an NMS decoder with $n_{E}=4$ and $n_{E}=5$, respectively. For all decoders, the channel and message quantization were set to be identical, i.e., $n_{E}=n_{Q}$. We used a different code for our implementation investigation than in the previous sections. This code has a larger block size, which implies increased implementation complexity. The code is a (816,406) regular LDPC code with $d_{V}=3$ and $d_{C}=6$ and the number of decoding iterations is I=8.

We applied a Synopsys Design Compiler and IC Compiler II for implementation in a 28 nm Fully-Depleted Silicon-on-Insulator (FD-SOI) technology under worst-case Process, Voltage and Temperature (PVT) conditions (125 °C, 0.9 V for timing, 1.0 V for power). A process with eight metal layers was chosen. Metal layers 1 to 6 are used for routing, with metals 1 and 2 mainly intended for standard cells. The metal layers 7 and 8 are only used for power supply. Power numbers were calculated with back-annotated wiring data and input data for a FER of $10^{-4}$. All designs were optimized for high throughput with a target frequency of 1 GHz during synthesis and back-end. To assess the routing congestion, we fixed the utilization to 70 % for all designs as a constraint. The utilization specifies the ratio between logic cell area and total area (=logic cell area plus routing area). Thus, by fixing this parameter, all designs have the same routing area available in relation to their logic cell area.

### 5.1. FER Performance of Implemented FA-MP Decoders

**Figure 10:**
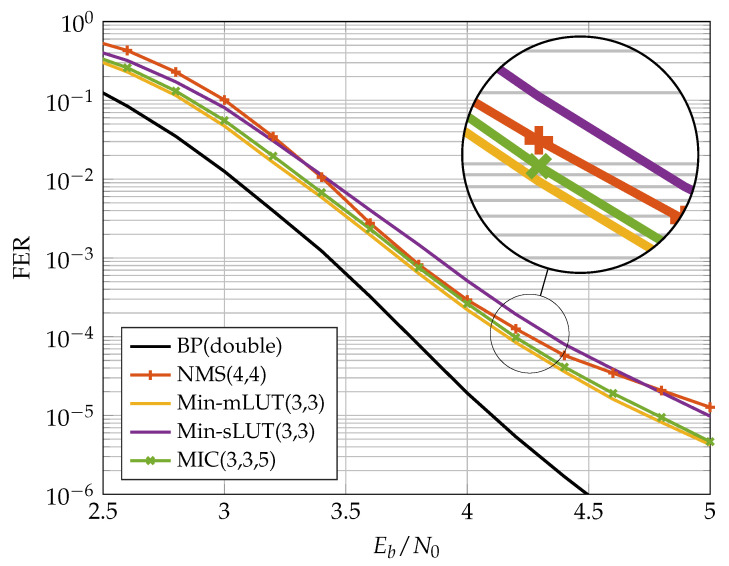
Communication performance of $n_{E}=3$ bit FA-MP decoders.

**Figure 11:**
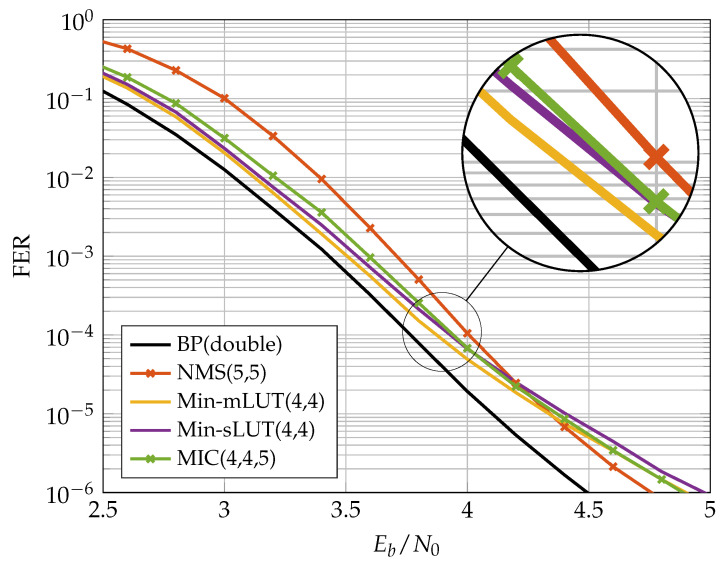
Communication performance of $n_{E}=4$ bit FA-MP decoders.

Figure 10 and Figure 11 show the FER performance for the different decoders. We compare the NMS decoder with the MIC decoder and the two LUT-based decoders. The LUTs of the FA-MP decoders are elaborated to a design Signal-to-Noise-Ratio (SNR) optimized at an FER of $10^{-4}$. It should be noted that this may result in an error floor behavior below the target FER. This phenomenon can be mitigated by selecting a larger design SNR at the cost of decreased performance in the waterfall region [13]. For comparison, we also added the BP performance with double precision floating point number representation.

In the previous section, we showed that, for the (648,486) code, the MIC decoder achieves the same error correction performance as the Min-mLUT decoder for $n_{R}=7$. A similar observation was made for the (816,406) code considered here. In our implementation comparison, we reduced $n_{R}$ such that the MIC’s FER stays below that of the NMS at the target FER of $10^{-4}$. In this way, we obtained an $n_{R}=5$, which yields a small degradation in the MIC FER compared to the Min-sLUT and Min-mLUT decoders, but outperforms the NMS decoder. We observe that the MIC and Min-mLUT decoders with one bit smaller message quantization $n_{E}$ have better error correction capability than the NMS decoder at the target FER. In addition, due to the low message quantization and the resulting low dynamic range, the NMS runs into an error floor below FER $10^{-4}$.

### 5.2. FD-SOI Implementation Results

Table 2 shows the implementation results for MIC(3,3,5), Min-mLUT(3,3), Min-sLUT(3,3) and NMS(4,4) decoders, whereas Table 3 shows the implementation results for MIC(4,4,5), Min-mLUT(4,4), Min-sLUT(4,4) and NMS(5,5) decoders. As already stated, we fixed the target frequency to 1 GHz and the utilization to 70% for all decoders. Maximum achievable frequency *f*, final utilization, area *A* and power consumption *P* were extracted from the final layout data. From these data, we can derive the important implementation metrics: throughput, latency, area efficiency and energy efficiency. Since the decoders are pipelined, the coded decoder throughput *T* is f·N. The latency is 1/f·26 (each iteration consists of three pipeline stages, decoder input and output are also buffered, yielding 8·3+2=26 pipeline stages in total). The area efficiency is defined as T/A and the energy efficiency as P/T.

The Min-mLUT decoder has the largest area, the worst area efficiency, and the worst energy efficiency. We see an improvement in these metrics for the Min-sLUT at the cost of a slightly decreased error correction performance. The difference in the implementation metrics largely increases when $n_{E}=3$ changes to $n_{E}=4$. The area increases by a factor of 10 for the Min-mLUT(4,4), but only by a factor of 2.7 for the Min-sLUT(4,4) decoder. Moreover, we had to reduce the utilization to 50 % to achieve a routing convergence for the MinmLUT(4,4) decoder. The large area increase is explainable with the increase of the LUT sizes from 512 to 4096 entries per LUT when increasing $n_{E}$ from 3 to 4. Moreover, the frequency largely breaks down, yielding a very low area efficiency and energy efficiency. The Min-sLUT decoders scale better with increasing $n_{E}$. Both Min-sLUT decoders outperform the corresponding NMS decoders in throughput and efficiency metrics.

The MIC decoder has the best implementation metric numbers in all cases. It outperforms all other decoders in throughput, area, area efficiency and energy efficiency while having the same or even slightly improved error correction performance compared to the other decoders. It can also be seen that the MIC decoder has a lower routing complexity compared to the Min-sLUT and the NMS decoder. We observe a large drop in the frequency from 595 MHz down to 183 MHz (70 % decrease) when comparing NMS(4,4) with NMS(5,5) under the utilization constraint of 70 %. The large drop in the frequency is explainable with the increased routing complexity for the given routing area constraint that yields longer wires and corresponding delays. This problem is less severe for the MinsLUT, where the frequency drops from 670 MHz to 492 MHz (27 % decrease). The MIC achieves the highest frequency for all cases and drops from 775 MHz to 633 MHz (18% decrease), only. This shows that the MIC scales much better with increasing $n_{E}$.

It should be noted that the CFU implementation is identical for the MIC, Min-mLUT and Min-sLUT decoders. Compared to the corresponding NMS, the CFU implementation is less complex [19] due to: (i) a 1 bit smaller message quantization, (ii) the omission of the scaling unit, and (iii) the omission of the sign-magnitude to two’s complement conversion. Hence, the CFU complexity of the FA-MP is always lower than that of the NMS independent of the respective CN degree. Moreover, in contrast to the NMS decoder, the messages from the CFUs to the VFUs are transmitted in sign-magnitude representation via the routing network which reduces the toggling rate and thus the average power consumption.

**Figure 12:**
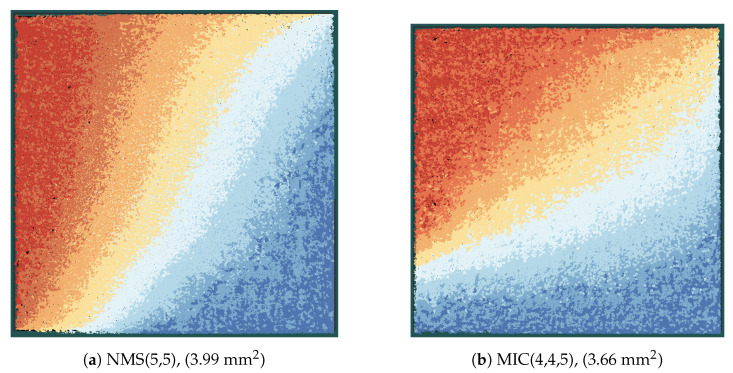
Layout of decoders in the same scale; each color indicates one iteration stage from dark red (first iteration) to dark blue (eighth iteration).

Figure 12 shows the layout of the MIC and the NMS decoder in the same scale. Each color represents one iteration stage, which is composed of CFUs, VFUs, and the routing between the nodes (see also Figure 9). When comparing the same iteration stages (same color) of the two decoders, we can observe that the iteration stages in the MIC decoder are smaller than the corresponding iteration stages in the NMS decoder, although the frequency of the MIC decoder is more than three times higher compared to the NMS decoder. This shows once again that the MIC has a lower implementation complexity, especially from a routing perspective.

Our analysis shows that the new MIC approach largely improves the implementation efficiency and exhibits better scaling compared to the state-of-the-art sLUT and NMS implementations of FP decoder architectures. This enables the processing of larger block sizes, which is mainly due to the reduced routing complexity. Larger block sizes improve the error correction capability and further increase the throughput of FP architectures.

## 6. Conclusions

This paper provides a detailed investigation of the Minimum-Integer Computation (MIC) decoder for regular and irregular Low-Density Parity Check (LDPC) codes. The MIC decoder utilizes the computational domain framework to realize Variable Node (VN) updates by an equivalent low-range signed integer computation and Check Node (CN) updates by a minimum search. For the VN update, we provide further insights for the design of an Mutual Information (MI) maximizing signed integer computation. To discuss implementation issues on FA-MP decoding architectures, we exemplified this on different LUT-based decoder designs. Furthermore, we compared MIC to state-of-the-art Normalized Min-Sum (NMS) decoder implementations to show the improvement in area efficiency and energy efficiency.

## Figures and Tables

**Table 1:** Numeric values of integer based VN update $g_{10}^{\mathrm{MIC}}$ with scaling parameter s=10.

Clusterindexz,a,t	±4	±3	±2	±1
Channel LLR L(z)	±5.07	±2.90	±1.56	±0.49
Integer mapping ϕ(z)	±51	±29	±16	±5
Message LLR L(a)	±3.46	±2.08	±1.02	±0.25
Integer mapping $\phi_{V}\left(a\right)$	±35	±21	±10	±3
Interval $W_{t}$	$\pm \left[121,42\right]$	$\pm \left[41,25\right]$	$\pm \left[23,12\right]$	$\pm \left[11,1\right]$

**Table 2:** Post-layout results of FA-MP decoders with $n_{E}=n_{Q}=3$, $n_{R}=5$.

	MIC	Min-mLUT	Min-sLUT	NMS
$n_{E}$, $n_{Q}$	3	3	3	4
$E_{b}/N_{0}$ @ FER $10^{-4}$ [dB]	4.20	4.16	4.35	4.26
Utilization [%]	70	68	71	71
Frequency [MHz]	775	662	670	595
Coded Throughput [Gb/s]	633	540	547	486
Area [mm^2^]	2.73	4.23	2.86	3.04
Area Efficiency [Gb/s/mm^2^]	231.6	128	190	159.7
Latency [ns]	33.5	39.3	35.8	43.7
Power [W]	4.49	5.07	4.38	4.39
Energy Efficiency [pJ/bit]	7.10	9.4	8.0	9.0

**Table 3:** Post-layout results of FA-MP decoders with $n_{E}=n_{Q}=4$, $n_{R}=5$.

	MIC	Min-mLUT	Min-sLUT	NMS
$n_{E}$, $n_{Q}$	4	4	4	5
$E_{b}/N_{0}$ @ FER $10^{-4}$ [dB]	3.94	3.87	3.93	4.01
Utilization [%]	69	49	66	69
Frequency [MHz]	633	267	492	183
Coded Throughput [Gb/s]	516	218	401	149
Area [mm^2^]	3.66	40.51	7.82	3.99
Area Efficiency [Gb/s/mm^2^]	141.1	5.4	51.3	37.4
Latency [ns]	41.1	97.2	48.0	142.0
Power [W]	5.61	11.85	8.68	2.25
Energy Efficiency [pJ/bit]	10.9	54.3	21.6	15.1

## Data Availability

Not applicable.
